# Epidemiology and Evolution of Bovine Viral Diarrhea Virus (BVDV) in Uruguay: A 10-Year Study

**DOI:** 10.3390/v17101374

**Published:** 2025-10-14

**Authors:** Leticia Maya, Matias Castells, Caroline Silveira, Federico Giannitti, Ingryd Merchioratto, Maria Barrandeguy, Alejo Menchaca, Rodney Colina

**Affiliations:** 1 Laboratorio de Virología, Departamento de Ciencias biológicas, CENUR Litoral Norte-Sede Salto, Universidad de la República, Gral. Rivera 1350, Salto 50000, Uruguay; matiascastellsbauer@gmail.com; 2 Plataforma de Investigación en Salud Animal, Estación Experimental La Estanzuela, Instituto Nacional de Investigación Agropecuaria (INIA), Ruta 50, km 11.5, La Estanzuela, Colonia 70006, Uruguay; cdasilvas@inia.org.uy (C.S.); fgiannitti@inia.org.uy (F.G.); imerchioratto@inia.org.uy (I.M.); mbarrandeguy@inia.org.uy (M.B.); amenchaca@inia.org.uy (A.M.)

**Keywords:** bovine viral diarrhea, BVDV, bovine viral diarrhea virus epidemiology, bovine viral diarrhea virus evolution, BVDV-1a, BVDV-2b, Uruguay

## Abstract

Bovine viral diarrhea virus (BVDV) is a pathogen of worldwide economic importance. In Uruguay, BVDV is endemic, with seroprevalence >80% at the farm level. This study analyzed 912 samples collected from January 2018 to October 2024 by reverse transcription PCR and sequencing, from calves with diarrhea, aborted fetuses, heifers with a history of abortions, and animals exhibiting symptoms of Mucosal Disease. This work summarizes ten years (2014–2024) of molecular epidemiology and evolution of BVDV. Analysis of the BVDV 5′UTR/N^pro^ genomic region revealed that the BVDV-1a, 1e, 1i, and 2b subtypes circulate in Uruguay. BVDV-1a remains the most prevalent subtype, followed by BVDV-2b, whose prevalence has been increasing. Our previous studies revealed that BVDV-1a showed geographical diversification in Uruguay. In this work, evolutionary studies conducted with N^pro^ genomic region showed that BVDV-2b is evolving at a substitution rate of 6.09 × 10^−4^ substitutions/site/year and has been introduced from Brazil in six separate events between 1870 and 1928, showing no geographical diversification. This work demonstrates that BVDV-1a and BVDV-2b are evolving differently in Uruguay. This evolutionary divergence is notable when comparing patterns observed in other countries where these subtypes circulate. Our findings provide crucial knowledge that should be considered for developing effective BVDV control measures in Uruguay.

## 1. Introduction

Bovine viral diarrhea virus (BVDV) is a widespread cattle pathogen with significant economic impact. It causes reproductive disorders such as embryonic death, abortions, and reduced fertility. The virus also affects livestock production by causing immunosuppression, respiratory problems, and diarrhea. Additionally, it creates persistently infected (PI) animals that are immunotolerant to the virus and continuously spread it through the herd [1,2,3].

BVDV belongs to the genus *Pestivirus* in the family *Flaviviridae*. It has a single-stranded, positive-sense RNA genome approximately 12.3 kb in length, with one open reading frame (ORF) that encodes a polyprotein of 3898 amino acid residues. This ORF is flanked at both ends by untranslated regions (UTRs) [4].

There are three BVDV species: BVDV-1, BVDV-2, and HoBi-like pestivirus (HoBiPev). They were recently renamed as *Pestivirus bovis*, *Pestivirus tauri*, and *Pestivirus brazilense*, respectively [5].

The BVDV-1 species is the most genetically diverse, with 23 viral subtypes described (BVDV-1a to BVDV-1w) [6,7,8,9,10,11,12,13].

In contrast, the BVDV-2 and HoBi-like pestivirus species are not as divergent. Five viral subtypes have been reported for each: BVDV-2a to BVDV-2e [14,15,16,17,18] and HoBi-like pestivirus subtypes a to e [11,19,20,21].

In the Americas, genetic diversity studies suggest that BVDV-1 and BVDV-2 have been circulating since the 1670s [22]. More recently, HoBiPev has emerged and appears to be disseminated in many regions of the world, especially in South America [22,23]. The BVDV species (BVDV-1, BVDV-2, and HoBiPev) are endemic to Latin America and are widely distributed globally, exhibiting varying levels of prevalence across different regions [24].

In Uruguay, there are approximately 12 million head of cattle, including both dairy and beef herds [25]. The presence of BVDV was first confirmed in 1996 through immunohistochemistry [26]. The virus is endemic in Uruguayan herds; according to data collected in 2015, herd-level seroprevalence was 98.8%, with values within individual farms of approximately 80% (Dr. Federico Fernández, Ministry of Livestock, Agriculture and Fisheries, MGAP, personal communication).

Currently, Uruguay has no national control program for BVDV, and vaccination is not mandatory. As a result, only 3% of producers vaccinate against the pathogen [27]. Yarnall and Thrusfield, (2017) estimated the economic impact of BVDV in endemic countries like Uruguay to be between USD8.4 and USD113 per cow per year [28].

BVDV is recognized as a significant pathogen that adversely affects reproductive efficiency in Uruguayan cattle herds [29].

Given the virus’s impact on Uruguayan cattle production, understanding its prevalence, molecular epidemiology, and the evolution of its species and subtypes is crucial for developing targeted control measures. Ten years ago, our group initiated work on BVDV, providing the first description of the species and subtypes circulating in Uruguayan herds. Our research included genetic analysis of these subtypes, the isolation of local strains, and the sequencing of their complete coding regions [30,31,32].

In this study, we present an updated overview of the molecular epidemiology of BVDV in Uruguay, along with a more in-depth evolutionary analysis. The results of this work will provide additional tools to strengthen BVDV prevention measures, develop appropriate control strategies for our herds, and thus reduce the economic losses it causes to the Uruguayan economy.

## 2. Materials and Methods

### 2.1. Sample Collection

A total of 912 serum and tissue samples were collected from January 2018 to October 2024. The samples were obtained from calves with diarrhea, aborted fetuses, heifers from farms with a history of abortions, or animals exhibiting symptoms of Mucosal Disease. All samples were submitted to the Virology Laboratory at the “Cenur Litoral Norte, Centro Universitario Salto, Universidad de la República” and stored at −20 °C until they could be tested.

Samples were obtained from INIA La Estanzuela, tissues were collected postmortem by veterinary pathologists from spontaneously aborted fetuses and/or cattle that died naturally, not requiring ethic committee approval. Sampling of serum obtained from live cattle by INIA La Estanzuela veterinarians was approved by INIA’s Ethics Committee for the Use of Animals in Experimentation (protocol number 2019.9).

### 2.2. RNA Extraction, Reverse Transcription and Sample Screening by Real-Time PCR

Viral RNA was extracted from all samples using the MagMAX Nucleic Acid Isolation Kit^®^ (Thermo Fisher Scientific) according to the manufacturer’s instructions. Reverse transcription of the RNA was performed using random primers and the RevertAid^®^ enzyme (Thermo Fisher Scientific, Waltham, MA, USA) following the manufacturer’s recommendations.

A real-time PCR assay was carried out to detect a 207 bp fragment of the 5′UTR genomic region of BVDV-1, BVDV-2, and HoBi-like pestivirus, as previously described by Maya et al. (2016, 2020) [30,31]. All real-time PCR reactions were performed using a SensiFAST II Probe Kit (Bioline Reagents Ltd., London, UK) and a Rotor-Gene Q instrument (QIAGEN), following the manufacturer’s recommendations.

### 2.3. Amplification by Conventional PCR

A 207 bp fragment of the 5′UTR was amplified from real-time PCR-positive samples, using the same primer pair used for the real-time PCR assay. Furthermore, a 428 bp fragment of the N^pro^ genomic region was also amplified as described by Maya et al. (2020) [31]. Positive fragments were purified using DNA Clean & Concentrator-5 (Zymo Research, Irvine, CA, USA), and Sanger sequenced at Macrogen, Inc., (Seoul, Republic of Korea).

### 2.4. Phylogenetic Analysis

Sequences were edited using SeqMan Software ULTRA version (DNASTAR Lasergene) and aligned with the Clustal W algorithm in MEGA X [33]. Genotype assignment was performed through phylogenetic analysis of a 607 bp concatenated fragment “5′UTR/N^pro^” composed of the Uruguayan 5′UTR and N^pro^ sequences along with representative BVDV-1, BVDV-2, and HoBi-like pestivirus sequences retrieved from the GenBank database. The concatenated fragment was created by combining the 5′UTR and N^pro^ sequences, which overlap by 28 bp.

Sequences from Uruguayan strains are listed in Table 1. Uruguayan BVDV strains previously published by Maya et al. (2016, 2020) [30,31] were also included in the analysis (n = 32) (Table 2). For samples in which only the 5′UTR genomic region was successfully amplified, subtyping was performed using the NCBI BLAST tool, with full awareness of its limitations in accurately resolving viral subtypes.

In phylogenetic analyses, a reference sequence of border disease virus (BDV) was used as an out-group (BDV X818). The model of nucleotide substitution that best fit the 5′UTR/N^pro^ dataset (GTR + gamma) was selected using the jModelTest program, based on the Akaike information criterion (AIC) [34]. The phylogenetic tree was then constructed using the maximum-likelihood (ML) method, and statistical significance was evaluated by the bootstrap method (1000 replicates) in MEGA X [33].

### 2.5. Population Dynamics Analysis of Uruguayan BVDV-2b

To investigate the evolutionary dynamics of BVDV-2b, we first explored the temporal structure of the sequences using TempEst v1.5.3, confirming it with a root-to-tip regression analysis [35].

We then jointly estimated the time of the most recent common ancestor (tMRCA), ancestral locations, and the evolutionary rate of the partial N^pro^ genomic region (374 bp) for the BVDV-2b sequences. Our dataset included 12 Uruguayan BVDV-2b strains along with 16 strains from Brazil, China, Slovakia, and USA, all of which were retrieved from the GenBank database.

The phylodynamic analysis was performed using BEAST v1.10.4 package [36], with an uncorrelated lognormal (UCLN) relaxed clock and a Bayesian Skyline coalescent model [37]. The nucleotide substitution model that best fit the dataset was TN93 + gamma.

Phylogeography was incorporated into the analysis using discrete sampling locations (countries) as a trait and applying Bayesian stochastic search variable selection [38]. The Markov chain Monte Carlo (MCMC) simulations were run for 100 million generations. Results were visualized using the Tracer v1.5.0 program, excluding the initial 10% of the run as burn-in. We accepted results with effective sample size (ESS) values higher than 200 for all parameters to ensure the convergence of the analysis.

## 3. Results

### 3.1. Genetic Diversity of Uruguayan BVDV Strains

A total of 48 samples tested positive for bovine viral diarrhea virus (BVDV) by real-time PCR. When combined with the 39 samples previously obtained [30,31], this brings the total number of BVDV-positive samples to 87 over the 2014–2024 period (Table 1).

Table 1 summarizes the sample names and viral species/subtypes. Supplementary Table S1 summarizes sample names, and GenBank accession numbers for the 5′UTR/N^pro^ and 5′UTR genomic regions.

Three of the 48 positive samples from this study could not be subtyped because the 5′UTR genomic region could not be amplified by PCR, leaving 45 subtypable samples.

Table 2 summarizes the genomic regions 5′UTR/N^pro^ and 5′UTR and total number of strains for each subtype. It includes data from the current study and a summary of strains previously reported by Maya et al. (2016, 2020) [30,31].

Of these, 12 samples were subtyped using the NCBI BLAST tool. This resulted in seven samples subtyped as BVDV-1a, three as BVDV-2b, one as BVDV-1i, and one as BVDV-1e (Table 1 and Table 2).

When combined with strains from previous studies (n = 5) [30,31], a total of 17 strains could be genotyped using the NCBI BLAST tool for their 5′UTR genomic region. The final breakdown for these 17 strains is 12 BVDV-1a, 1 BVDV-1e, 1 BVDV-1i and 3 BVDV-2b (Table 1 and Table 2).

For the remaining 33 positive samples, the 5′UTR and N^pro^ genomic regions were successfully amplified, sequenced, and edited. The concatenated 5′UTR/N^pro^ fragments were subsequently subtyped through phylogenetic analysis.

A total of 67 strains were used for phylogenetic analysis in this work, consisting in 33 newly subtyped samples and 32 strains from previous studies [30,31], and two additional strains (3664UYCNIA/2017 and 3665UYCNIA/2017) from Maya et al. (2020) [31] for which the 5′UTR/N^pro^ genomic region was successfully amplified (Table 2).

**Figure 1:**
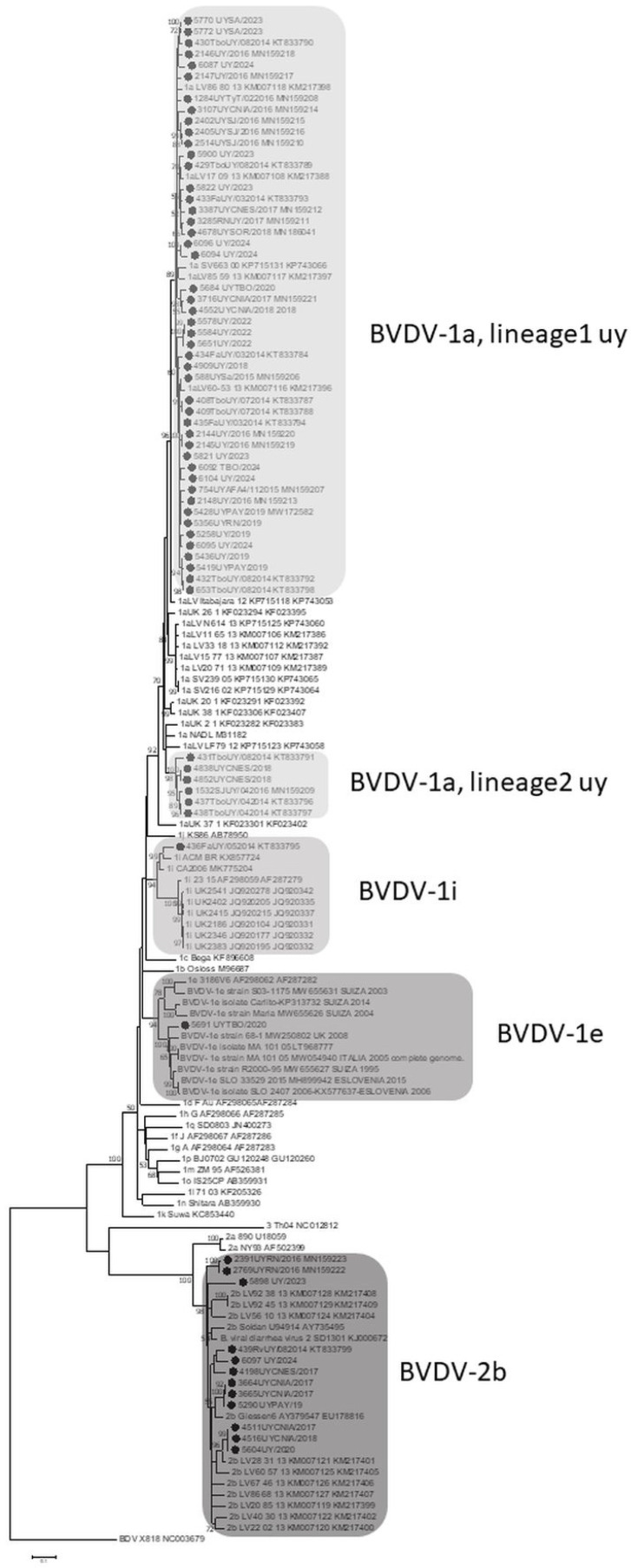
Phylogenetic analysis of BVDV strains based on the 5′UTR/N^pro^ genomic region. Uruguayan strains are indicated by black dots. The BVDV-1a, BVDV-1e, BVDV-1i, and BVDV-2b clades are highlighted in gray and indicated on the right side of the figure. Numbers at the tree branches represent bootstrap values. A border disease virus (BDV) sequence was included in the analysis as an out-group.

The genotyping of the 67 positive samples was performed using phylogenetic analysis based on a 607 bp fragment of the 5′UTR/N^pro^ region (Figure 1). The analysis revealed that 55 Uruguayan strains belonged to the BVDV-1 species, with 100% bootstrap support, while the remaining 12 strains belonged to the BVDV-2 species, also with 100% bootstrap support (Figure 1).

Within the BVDV-1 species, three subtypes were identified: BVDV-1a was the most prevalent with 53 samples, followed by BVDV-1i and BVDV-1e, with one sample each. All 12 strains of the BVDV-2 species were subtyped as BVDV-2b.

Phylogenetic analysis showed the two Uruguayan BVDV-1a clades previously described by our group. The main clade, BVDV-1a lineage 1 UY, was composed of 47 BVDV-1a sequences. This lineage formed a group with Brazilian strains, supported by an 89% bootstrap value. In contrast, the BVDV-1a lineage 2 UY, with 98% bootstrap support, was formed by the remaining six Uruguayan sequences (Figure 1).

The Uruguayan strain 436FaUY/052014 subtyped as BVDV-1i [30] clustered with the Brazilian strain ACM/BR/2016 and the USA strain CA2006 (99% bootstrap), as well as with strains from the United Kingdom (94% statistical support). The Uruguayan strain 5691 UYTBO/2020 was subtyped as BVDV-1e because it grouped with European strains of that viral subtype with 94% statistical support (Figure 1).

BVDV-2b Uruguayan strains were divided into five clades. Strains 2391UYRN/2016 and 2769UYRN/2016 formed a cluster with a 100% bootstrap value, separating from the other 10 strains. These remaining strains were divided into four clades, grouping with Brazilian strains of this subtype (Figure 1).

### 3.2. Population Dynamics Analysis of Uruguayan BVDV-2b Strains

**Figure 2:**
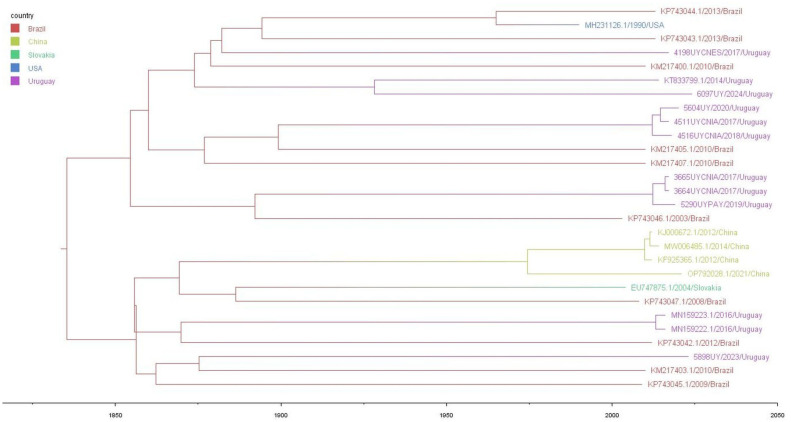
Phylogeographic analysis. This maximum clade credibility tree shows the geographic origin of the BVDV-2b strains. Branches are colored by country of origin: Brazil (red), China (light green), Slovakia (green), USA (blue), and Uruguay (lilac).

Our analysis revealed that the BVDV-2b subtype emerged in Brazil in 1835 (1475–1951, 95% HPD) and evolved at a substitution rate of 6.09 × 10^−4^ substitutions/site/year (1.23 × 10^−3^–1.01 × 10^−4^, 95% HPD). Subsequently, two lineages arose in Brazil and spread to other countries, including Uruguay. The phylogeographic analysis revealed six clades of Uruguayan BVDV-2b, indicating six separate introductions of this subtype from Brazil between 1870 and 1928 (Figure 2).

## 4. Discussion

Uruguayan economy is significantly driven by its agroindustrial sector, with exports representing 71.9% of the country’s total exports. Livestock production plays a central role; in 2023, approximately 25.6% of Uruguay’s exports consisted of beef and live cattle, generating around USD 2.3 million [25].

BVDV is endemic throughout Uruguay, with seroprevalence values of around 80% (Dr. Federico Fernández, MGAP, personal communication). Successfully BVDV control strategies have been implemented in countries such as Scotland, New Zealand, and Switzerland. These programs are typically funded on continuous virus surveillance, vaccination, and the culling of persistently infected (PI) animals [39]. Such measures have proven effective mitigating economic losses caused by the pathogen [40].

Uruguay lacks a national health program for BVDV control, and vaccination is not mandatory, only 3% of producers vaccinate against the pathogen [27]. Additionally, previous studies have shown that two amino acid residue substitutions in the E2 glycoprotein of Uruguayan BVDV-1a strains, relative to the vaccine strain NADL, could potentially compromise vaccine efficacy [31].

Recognizing BVDV significant impact on Uruguayan herds this current study expands upon the previous sampling and supports the molecular epidemiology data. From 2014 to 2024, a total of 3.458 samples were analyzed for BVDV, with 87 samples testing positive by Real Time PCR for the virus. These samples were obtained from herds with reproductive problems, calves with diarrhea, aborted fetuses, heifers from farms with a history of abortions, and animals with Mucosal disease symptoms.

We found that 69 of the BVDV positive strains (82%) belonged to the species BVDV-1, while the remaining 15 (18%) belonged to the species BVDV-2 (Table 2). Based on our sampling, HoBiPev does not appear to be circulating in our herds. These results contrast with our bordering countries, Brazil and Argentina. Both countries, like Uruguay, have significant bovine production, but all three BVDV species are present in their herds and show greater diversity of BVDV subtypes [41,42,43,44,45,46,47,48]. However, it is important to note that the sampling in this study was not nationwide and included only symptomatic animals, excluding asymptomatic individuals. Therefore, the findings may not fully reflect the circulating subtypes or their true prevalence.

Our findings indicate that the BVDV-1a subtype remains the most frequent subtype in our country, accounting for 77.4% (n = 65) of the analyzed strains (Table 2). The phylogenetic tree (Figure 1) showed that Uruguayan BVDV-1a strains are still evolving and continue to show geographical diversification into two distinct lineages: “BVDV-1a lineage 1 UY” and “BVDV-1a lineage 2 UY,” consistent with our previous work [31].

The BVDV-1i subtype represented 2.3% (n = 2) of the positive samples (Table 2). Although this subtype was first described in the United Kingdom in 1999 [49] and initially appeared to be restricted to that country, its presence was subsequently detected worldwide. A complete genome from the USA, submitted to GenBank in 2019, dates back to 2006 (CA2006, accession number to GenBank database MK775204). It was later found in Uruguay in 2014 (Maya et al., 2016) and in Brazil in 2016 [50]. The strains from the USA, Brazil, and Uruguay (436FaUY/052014) grouped together with 99% statistical support, forming a distinct cluster from those found in the United Kingdom (Figure 1).

Uruguayan strains 5688UYTBO/2020 and 5691 UYTBO/2020 represent the first description of the BVDV-1e subtype in Uruguay and accounted for 2.3% (n = 2) of the positive samples (Table 2). This subtype appears to be highly prevalent in Europe [51]. Although strain LV/LF15/12 was detected in Brazil in 2012 [45], it could not be included in our phylogenetic analysis because only its 5′UTR genomic region was available on the GenBank database. Uruguayan strain (5691 UYTBO/2020) was closely related to the European strains (Figure 1).

It is true that BVDV-2 is not common in South America, but BVDV-2b is a highly prevalent subtype in Brazil [51]. The prevalence of this subtype has increased in our country over the years to 18% of positive samples (n = 15) (Table 2). The increasing prevalence of the BVDV-2b subtype in our herds over the years supports the recommendation to include this subtype in future vaccine formulations. As with our previous work [31], Uruguayan strains are closely related to Brazilian ones (Figure 1).

A deeper evolutionary analysis of Uruguayan BVDV-2b strains using the N^pro^ genomic region and sequences available on GenBank revealed that the BVDV-2b subtype emerged in Brazil in 1835 and evolved at a substitution rate of 6.09 × 10^−4^ substitutions/site/year (1.23 × 10^−3^–1.01 × 10^−4^, 95% HPD). It gave rise to two lineages that spread to other countries, including Uruguay. Our results indicate that the BVDV-2b subtype entered Uruguay from Brazil on six separate occasions between 1870 and 1928 (Figure 2). The wide HPD intervals observed in the substitution rate estimations (10^−3^–10^−4^) are likely influenced by the limited dataset used in this study (n = 12 from Uruguay). Additionally, the scarcity of BVDV-2b N^pro^ genomic sequences with complete metadata (e.g., origin and collection date) available in the GenBank database (n = 16) represents a limitation for robust phylogenetic inferences. This study represents an initial understanding the evolutionary dynamics of BVDV-2b in Uruguay. Further research incorporating a larger number of BVDV-2b sequences from both Uruguay and other regions is needed to improve the precision of HPD intervals and yield more reliable evolutionary estimates.

Although the branch lengths observed in the phylogeographic analysis for each entry event are long, the introductions of this subtype to Uruguay appear to be recent. This could be attributed to a lack of strain sampling between the root of each entry and the year of sample collection. Furthermore, BVDV-2b does not show geographical diversification after each introduction and evolves more slowly than BVDV-1a [31]. This evolutionary pattern contrasts with BVDV-1a’s geographical diversification in Uruguay, where the phylogenetic branches are shorter and Uruguayan strains is a descendant from a local ancestor [31]. BVDV-1a and BVDV-2b appear to exhibit distinct evolutionary dynamics over time, and does not appear to be related to vaccination, likely to its limited (3% of the farmers vaccinate) and non-mandatory use. These differences may be attributed to varying selective pressures acting on each subtype, potentially influenced by underlying biological factors. Another plausible explanation is that BVDV-1a possesses greater fitness, enabling more efficient transmission within local cattle herds. Nonetheless, further research is necessary to substantiate this hypothesis.

In contrast to the evolutionary patterns observed in Uruguay for BVDV-1a and BVDV-2b, studies in the United Kingdom and Brazil show different behaviors. In the United Kingdom, where BVDV-1a is also predominant, small clusters of isolates from the same province are generally from the same farm. This suggests farm-specific grouping rather than wider geographical diversification [52]. Conversely, in Brazil, where BVDV-2b is the predominant BVDV-2 subtype, strains of this subtype cluster together, indicating geographical diversification [45].

In summary, this work provides an updated overview of BVDV in Uruguay over a ten-year period. It updates the molecular epidemiology in Uruguayan herds, provides a deeper description of the evolutionary patterns of BVDV-1a and BVDV-2b, and discusses how their evolution differs from each other and that in other countries. The findings of this study underscore the necessity of developing vaccines that incorporate locally circulating BVDV-1a and BVDV-2b subtypes in their formulation. Furthermore, the data generated herein offer critical insights to inform the design and implementation of targeted and effective BVDV control strategies within the Uruguayan context.

## Figures and Tables

**Table 1:** This table summarizes the strains analyzed in this study and those reported by Maya et al. (2016, 2020) [30,31]. It includes the sample name and viral species/subtypes.

Sample Name	BVDV Species/Subtype	
408TboUY/072014	BVDV-1a	1
409TboUY/072014	BVDV-1a	2
429TboUY/082014	BVDV-1a	3
430TboUY/082014	BVDV-1a	4
431TboUY/082014	BVDV-1a	5
432TboUY/082014	BVDV-1a	6
433FaUY/032014	BVDV-1a	7
434FaUY/032014	BVDV-1a	8
435FaUY/032014	BVDV-1a	9
437TboUY/042014	BVDV-1a	10
438TboUY/042014	BVDV-1a	11
653TboUY/082014	BVDV-1a	12
651TboUY/082014	BVDV-1a	13
652UYTbo/082014	BVDV-1a	14
588UYSa/2015	BVDV-1a	15
754UYAFA4/112015	BVDV-1a	16
1284UYTyT/022016	BVDV-1a	17
1532SJUY/042016	BVDV-1a	18
2144UY/2016	BVDV-1a	19
2145UY/2016	BVDV-1a	20
2146UY/2016	BVDV-1a	21
2147UY/2016	BVDV-1a	22
2148UY/2016	BVDV-1a	23
2402UYSJ/2016	BVDV-1a	24
2405UYSJ/2016	BVDV-1a	25
2514UYSJ/2016	BVDV-1a	26
3107UYCNIA/2016	BVDV-1a	27
3285RNUY/2017	BVDV-1a	28
3387UYCNES/2017	BVDV-1a	29
3397UYCNES/2017	BVDV-1a	30
3716UYCNIA/2017	BVDV-1a	31
3723UYCNIA/2017	BVDV-1a	32
3738UYLAV/2017	BVDV-1a	33
4552UYCNIA/2018	BVDV-1a	34
4678UYSOR/2018	BVDV-1a	35
4838UYCNES/2018	BVDV-1a	36
4852UYCNES/2018	BVDV-1a	37
4909UY/2018	BVDV-1a	38
5160 UYPAY/19	BVDV-1a	39
5258UY/2019	BVDV-1a	40
5354UYRN/2019	BVDV-1a	41
5356UYRN/2019	BVDV-1a	42
5419UYPAY/2019	BVDV-1a	43
5428UYPAY/2019	BVDV-1a	44
5436UY/2019	BVDV-1a	45
5578UY/2022	BVDV-1a	46
5582UYCNIA/2022	BVDV-1a	47
5584UY/2022	BVDV-1a	48
5615UY/2020	BVDV-1a	49
5651UY/2022	BVDV-1a	50
5684 UYTBO/2020	BVDV-1a	51
5685 UYTBO/2020	BVDV-1a	52
5767 UYSA/2023	BVDV-1a	53
5770 UYSA/2023	BVDV-1a	54
5772 UYSA/2023	BVDV-1a	55
5821 UY/2023	BVDV-1a	56
5822 UY/2023	BVDV-1a	57
5900 UY/2023	BVDV-1a	58
6067 UY/2024	BVDV-1a	59
6087 UY/2024	BVDV-1a	60
6092 TBO/2024	BVDV-1a	61
6094 UY/2024	BVDV-1a	62
6095 UY/2024	BVDV-1a	63
6096 UY/2024	BVDV-1a	64
6104 UY/2024	BVDV-1a	65
436FaUY/052014	BVDV-1i	66
5495UY/2019	BVDV-1i	67
5688UYTBO/2020	BVDV-1e	68
5691 UYTBO/2020	BVDV-1e	69
439RvUY/082014	BVDV-2b	70
2391UYRN/2016	BVDV-2b	71
2769UYRN/2016	BVDV-2b	72
3664UYCNIA/2017	BVDV-2b	73
3665UYCNIA/2017	BVDV-2b	74
4198UYCNES/2017	BVDV-2b	75
4511UYCNIA/2017	BVDV-2b	76
4516UYCNIA/2018	BVDV-2b	77
5280UY/2019	BVDV-2b	78
5281UY/2019	BVDV-2b	79
5282UY/2019	BVDV-2b	80
5290 UYPAY/19	BVDV-2b	81
5604UY/2020	BVDV-2b	82
5898 UY/2023	BVDV-2b	83
6097 UY/2024	BVDV-2b	84
5905 UY/2024	not genotyped	85
5690UYTBO/2020	not genotyped	86
5693 UYTBO/2020	not genotyped	87

**Table 2:** This table summarizes the genomic regions 5′UTR/N^pro^, and 5′UTR, and total number of strains for each subtype (bold highlight). It includes data from the current study and a summary of strains previously reported by Maya et al. (2016, 2020) [30,31].

		Subtype	5UTR/N^pro^	5UTR	Total
		BVDV-1a	28	5	33
		BVDV-1i	1	0	1
		BVDV-2b	3	2	5
Maya et al., 2016, 2020	**Total**		**32**	**7**	**39**
		BVDV-1a	25	7	32
		BVDV-1e	1	1	2
		BVDV-1i	0	1	1
		BVDV-2b	7	3	10
This study	**Total**		**33**	**12**	**45**
		BVDV-1a	53	12	65
		BVDV-1e	1	1	2
		BVDV-1i	1	1	2
		BVDV-2b	12 *	3 *	15
Maya et al., 2016, 2020 + this study	**Total**		**67**	**17**	**84**

* Strains 3664UYCNIA/2017 and 3665UYCNIA/2017 (Maya et al., 2020) [30,31] were included, for which the 5′UTR/ N^pro^ genomic region was successfully amplified; the 5′UTR alone was excluded.

## Data Availability

GenBank accession numbers for the 5′UTR/N^pro^ and 5′UTR genomic regions: PX240601-PX240645 (Table S1).

## Supplementary Materials

The following supporting information can be downloaded at: https://www.mdpi.com/article/10.3390/v17101374/s1 Table S1: This table summarizes sample names, and GenBank accession numbers for the 5’ UTR/N^pro^ and 5’UTR genomic regions.
