# Supplementary material for: Epidemiology and Evolution of Bovine Viral Diarrhea Virus (BVDV) in Uruguay: A 10-Year Study
Source: Viruses. 2025 Oct 14;17(10):1374. doi: 10.3390/v17101374 (PMC12567747; doi:10.3390/v17101374)
Supplement: Supplementary file 1 [file viruses-17-01374-s001.zip › viruses-3874259-supplementary.pdf]

Supplementary Table S1. This table summarizes sample names, and GenBank accession numbers for the 5′ UTR/N^{pro} and 5′UTR genomic regions.

sample name	acc number 5`UTR/Npro	acc number 5`UTR
408TboUY/072014	KT833787	
409TboUY/072014	KT833788	
429TboUY/082014	KT833789	
430TboUY/082014	KT833790	
431TboUY/082014	KT833791	
432TboUY/082014	KT833792	
433FaUY/032014	KT833793	
434FaUY/032014	KT833784	
435FaUY/032014	KT833794	
437TboUY/042014	KT833796	
438TboUY/042014	KT833797	
653TboUY/082014	KT833798	
651TboUY/082014		KT833785
652UYTbo/082014		KT833786
588UYSa/2015	MN159206	
754UYAFA4/112015	MN159207	
1284UYTyT/022016	MN159208	
1532SJUY/042016	MN159209	
2144UY/2016	MN159220	
2145UY/2016	MN159219	
2146UY/2016	MN159218	
2147UY/2016	MN159217	
2148UY/2016	MN159213	
2402UYSJ/2016	MN159215	
2405UYSJ/2016	MN159216	
2514UYSJ/2016	MN159210	
3107UYCNIA/2016	MN159214	
3285RNUY/2017	MN159211	
3387UYCNES/2017	MN159212	
3397UYCNES/2017		MN159203
3716UYCNIA/2017	MN159221	
3723UYCNIA/2017		MN159202
3738UYLAV/2017		MN159201
4552UYCNIA/2018	PX240613	
4678UYSOR/2018	MN186041	
4838UYCNES/2018	PX240614	
4852UYCNES/2018	PX240615	
4909UY/2018	PX240616	
5160 UYPAY/19		PX240601

5258UY/2019	PX240617	
5354UYRN/2019		PX240602
5356UYRN/2019	PX240618	
5419UYPAY/2019	PX240619	
5428UYPAY/2019	MW172582	
5436UY/2019	PX240620	
5578UY/2022	PX240621	
5582UYCNIA/2022		PX240603
5584UY/2022	PX240622	
5615UY/2020		PX240604
5651UY/2022	PX240623	
5684 UYTBO/2020	PX240624	
5685 UYTBO/2020		PX240605
5767 UYSA/2023		PX240606
5770 UYSA/2023	PX240625	
5772 UYSA/2023	PX240626	
5821 UY/2023	PX240627	
5822 UY/2023	PX240628	
5900 UY/2023	PX240629	
6067 UY/2024		PX240607
6087 UY/2024	PX240630	
6092 TBO/2024	PX240631	
6094 UY/2024	PX240632	
6095 UY/2024	PX240633	
6096 UY/2024	PX240634	
6104 UY/2024	PX240635	
436FaUY/052014	KT833795	
5495UY/2019		PX240609
5688UYTBO/2020		PX240608
5691 UYTBO/2020	PX240636	
439RvUY/082014	KT833799	
2391UYRN/2016	MN159223	
2769UYRN/2016	MN159222	
3664UYCNIA/2017	PX240637	
3665UYCNIA/2017	PX240638	
4198UYCNES/2017	PX240639	
4511UYCNIA/2017	PX240640	
4516UYCNIA/2018	PX240641	
5280UY/2019		PX240610
5281UY/2019		PX240611
5282UY/2019		PX240612
5290 UYPAY/19	PX240642	
5604UY/2020	PX240643	
5898 UY/2023	PX240644	

6097 UY/2024
5905 UY/2024
5690UYTBO/2020
5693 UYTBO/2020

PX240645
